# Vitamin D Deficiency in the Gulf Cooperation Council: Exploring the Triad of Genetic Predisposition, the Gut Microbiome and the Immune System

**DOI:** 10.3389/fimmu.2019.01042

**Published:** 2019-05-10

**Authors:** Parul Singh, Manoj Kumar, Souhaila Al Khodor

**Affiliations:** Research Department, Sidra Medicine, Doha, Qatar

**Keywords:** hypovitaminosis D, microbial dysbiosis, VDR, VDBP, CYP27B1, GCC

## Abstract

Vitamin D is a fat soluble secosteroid that is primarily synthesized in the skin upon exposure to Ultraviolet B (UVB) sun rays. Vitamin D is essential for the growth and development of bones and helps in reducing inflammation by strengthening muscles and the immune system. Despite the endless supply of sunlight in the Gulf Cooperation Council (GCC) countries which includes United Arab Emirates, Qatar, Kuwait, Bahrain, Saudi Arabia, and Oman, Vitamin D deficiency in the (GCC) general population at various age groups remains alarmingly high. In parallel runs the increasing prevalence of acute and chronic illnesses including, autoimmune diseases, cancer, type 1 diabetes mellitus, cardiovascular disease and Inflammatory bowel disease in the adult as well as the pediatric population of these countries. The exact association between Vitamin D deficiency and chronic disease conditions remains unclear; however, studies have focused on the mechanism of Vitamin D regulation by assessing the role of the Vitamin D associated genes/proteins such as VDR (Vitamin D receptor), VDBP (Vitamin D Binding protein), CYP27B1 as these are integral parts of the Vitamin D signaling pathway. VDR is known to regulate the expression of more than 200 genes across a wide array of tissues in the human body and may play a role in controlling the Vitamin D levels. Moreover, reduced Vitamin D level and downregulation of VDR have been linked to gut dysbiosis, highlighting an intriguing role for the gut microbiome in the Vitamin D metabolism. However, this role is not fully described yet. In this review, we aim to expand our understanding of the causes of Vitamin D deficiency in the GCC countries and explore the potential relationship between the genetic predisposition, Vitamin D levels, immune system and the gut microbiome composition. Trying to unravel this complex interaction may aid in understanding the mechanism by which Vitamin D contributes to various disease conditions and will pave the way toward new therapeutics treatments for Vitamin D deficiency and its associated outcomes.

## Introduction

Vitamin D is well-known for its many health benefits. The role of Vitamin D in the elimination of rickets remains one of the most notable discoveries in medicine (1). Calcium and phosphate are two minerals that are required for normal bone formation. Upon demand, Vitamin D stimulates the intestines, bones, and kidneys to maintain calcium and phosphorus levels in the blood, and thus promotes the mineralization of the bone matrix and osteoclasts differentiation (2). Hypovitaminosis D or severe Vitamin D deficiency results in poor mineralization and bone loss, leading to osteoporosis, fractures, muscle weakness, and frank hypocalcemia (3). Vitamin D mediates its biological function via metabolizing into its active steroid form 1α,25-dihydroxyvitamin D3 [1α,25(OH)_2_D_3_ or simply 1,25(OH)_2_D], often referred to as a hormone, influencing many genes across various tissues in the human body, such as kidneys (4–6), intestines (7), bones (8–11), as well as cancer and immune cells (12–14).

Vitamin D is also known to interact with cells from both the innate and adaptive immune system, where it plays an important role in antigen presentation, immune regulation and antibacterial response (15). Historical Vitamin D supplementation has been used to treat lupus and mycobacterial infections such as tuberculosis and leprosy (16, 17). Studies examining the immunomodulatory properties of Vitamin D have linked its deficiency to a higher incidence of autoimmune diseases, such as type I diabetes (T1D), multiple sclerosis (MS), systemic lupus erythematosus (SLE), rheumatoid arthritis (RA), and inflammatory bowel disease (IBD) and also to an increased risk of developing various types of cancers including breast, ovarian, colon, and prostate cancer (18–21). Vitamin D is also a potent effector of vascular endothelial cells and thus has a role in cardio-protection (22), as it regulates blood pressure by stimulating the renin angiotensin system, vascular calcification and smooth muscle cell proliferation (23). Hypovitaminosis D is directly linked to the development of hypertension, and a reduction in blood pressure has been noted to be a consequence of Vitamin D supplementation (24, 25). Evidence also supports the role of Vitamin D in neuroprotection, brain development and maintenance of cognitive functions via immunomodulation, neuronal calcium regulation, antioxidative mechanisms, enhanced nerve conduction, and detoxification mechanisms (26). There have been some reports linking decreased Vitamin D levels with depression (27), cognitive delays and an increased risk of Alzheimer's disease (28). Cerebrovascular events such as the risk of vessel thrombosis, cerebrovascular infracts and strokes have also been associated with deficiency of Vitamin D (29).

Insufficient levels of Vitamin D may also be a contributing factor for other abnormalities such as poor diet, short stature, liver disease, and diabetes (30–32). The role of Vitamin D during pregnancy is also of great consideration since maternal nutritional status determines the health of fetus and newborn. Maternal Vitamin D deficiency has been associated with increased risk of preeclampsia, calcium malabsorption, bone loss, and other myopathies (33). It is suggested that the development of the fetus in a state of hypovitaminosis D can have significant impact on innate immune functions. *In vitro* study conducted with Monocytes cultured in Vitamin D deficient plasma, showed significantly decreased in a TLR-dependent expression of cathelicidin compared to the control (34) Vitamin D status in the cord blood was found to be associated with the risk of lower respiratory tract infection in the first year of life consistent with the *in vitro* study results (35). Severe deficiency can also contribute toward abnormalities such as small stature for gestational age, neonatal hypocalcemia, hypocalcemic seizures, infantile heart failure, enamel defects, large fontanelle, congenital rickets, among others (36, 37). The role of Vitamin D goes above and beyond the traditionally ascribed ones and its significance in human physiology is undeniable. In the most recent years, several papers have addressed the importance of Vitamin D and its intracellular receptor VDR in regulation of gut hemostasis and immune response (38–41), here we aim to go further and present a comprehensive review examining the epidemic of Vitamin D deficiency in Gulf Cooperation Council (GCC) which is an alliance of six Middle Eastern countries—Saudi Arabia, Qatar, Kuwait, the United Arab Emirates, Bahrain, and Oman. We would also like to study the possible role of specific genes in predisposing the gulf population to Vitamin D deficiency, and how this increasing epidemic leads to disturbed microbial balance in the intestines and manifestation of various immune mediated diseases such as IBD.

## Methods Used to Review the Literature in the Field

A comprehensive literature search was carried out in PubMed, ScienceDirect, Google Scholar and SpringerLink databases using keywords like “Vitamin D,” “Human microbiome,” “Vitamin D metabolism,” “Vitamin D and Gut microbiome,” “Vitamin D deficiency in Gulf countries,” “VDR and Immune regulation,” “Immune related disease in Gulf countries,” “25-hydroxy vitamin D.” Only articles published in English and related to the study topic were included in this review. The search was not restricted to the type of study i.e., species, meta-analysis, case-control, randomized control trials, cohort studies, reviews, sample size, or year of publication. Bibliographies and citation of the included reviews were scanned for additional studies that may have been missed by the database searches. The exclusion criteria comprised the following: unpublished data, conference publications, articles available only in the abstract form, and doctoral or master's thesis. Endnote software (Thomas Reuters, Philadelphia, PA) was used to create library and manage the findings of the search as recognized by the above-mentioned strategies. The selected articles were read and organized under the following headings: (1) Vitamin D, importance, metabolism, status and supplementation (2) Prevalence of Vitamin D deficiency or insufficiency and immune related disorders in GCC countries (3). Possible relationship between disturbances of the gut microbiota and/or Vitamin D deficiency, VDR dysfunction, and role of Vitamin D in immune system and related disorders.

## Vitamin D Metabolism

**Figure 1:**
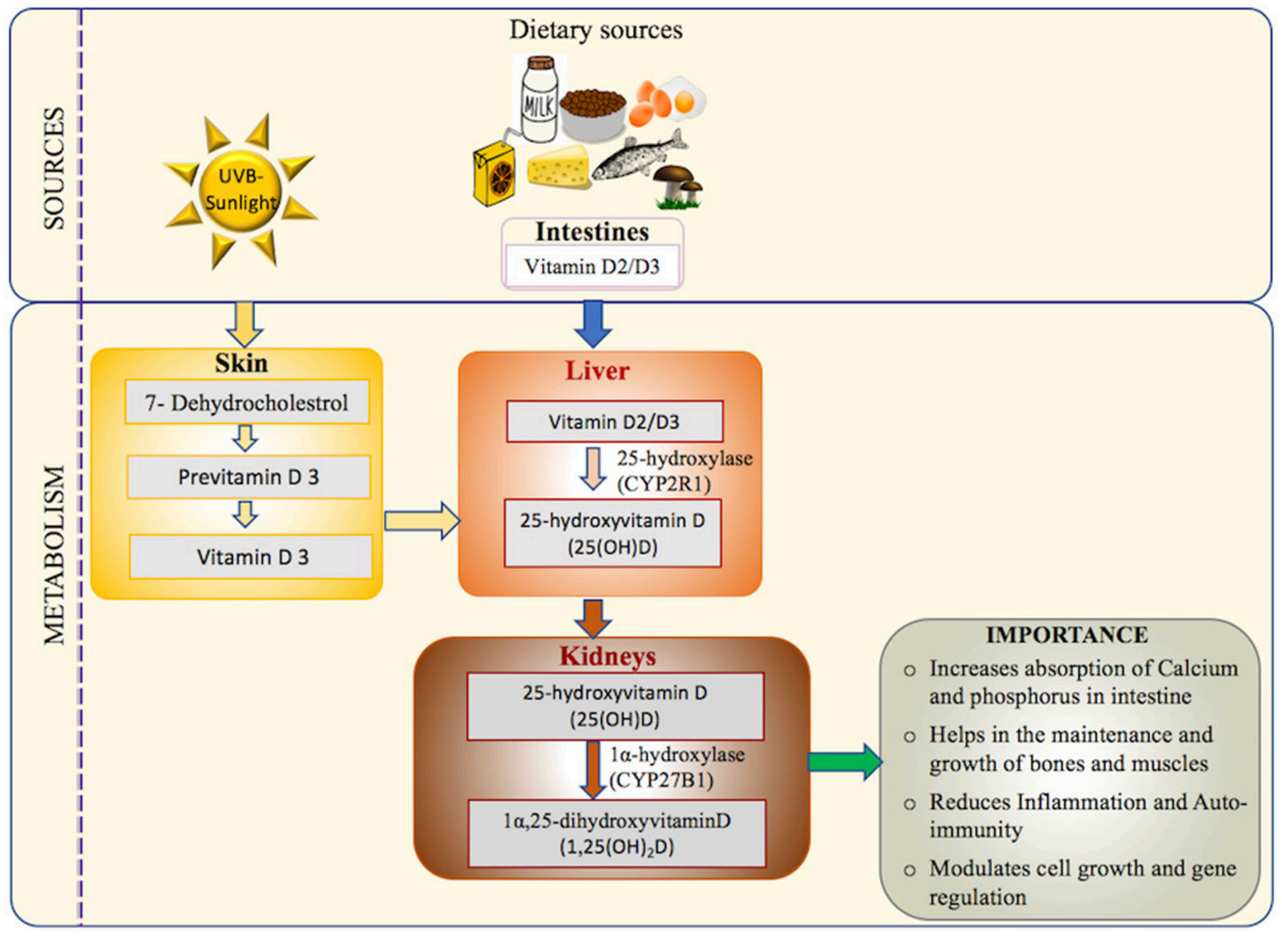
Vitamin D: sources, activation pathway, and importance in the human body: the major source of Vitamin D (UVB-sunlight) and the minor source (Dietary Vitamin D) are transported to liver and metabolized to its main circulating form (25-hydroxyvitamin D) which is measured in the serum in most assays (18, 42, 43). The activated form of Vitamin D is then synthesized in the kidney via hydroxylation to form 1,25-dihydroxyvitamin D, also known as calcitriol (44, 45). Calcitriol has many functions including mineralization of bone matrix, enhancing absorption of calcium and phosphorous from small intestines, reducing autoimmunity and inflammation, and gene regulation (20, 46).

In humans, Vitamin D originates from three potential sources: Ultraviolet B (UVB) dependent exogenous synthesis, nutritional sources and supplements (18, 42, 43). However, it is primarily synthesized in the skin by the action of sunlight (UV dependent) as very few naturally occurring food sources have adequate amounts of Vitamin D (18, 42, 43). Dietary sources such as fish, milk, orange juice, and cereals contain one of the two forms, cholecalciferol (Vitamin D_3_) or ergocalciferol (Vitamin D_2_) (18, 42, 43). Regardless of the source, the Vitamin D synthesis pathway follows several common steps as detailed in Figure 1.

**Figure 2:**
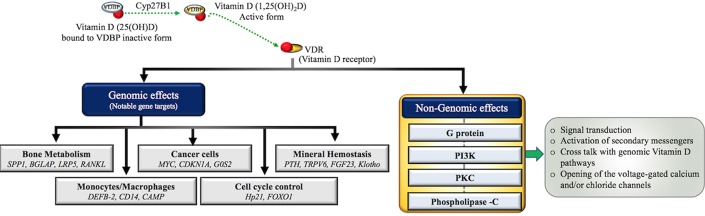
Molecular actions of the Vitamin D/VDR axis. Inactive Vitamin D (25(OH)D) circulates in the blood stream bound to Vitamin D binding protein (VDBP) and undergoes hydroxylation by the renal mitochondrial 1-hydroxylase (CYP27B1) enzyme to convert into active form (1α,25(OH)_2_D). Active Vitamin D binds to its primary receptor VDR to modulate the expression of more than 200 genes in human body. Some notable ones are grouped into various biological processes including (1) bone metabolism (8–11) (2) immune cell regulation (14, 49) (3) cancer (12) (4) cell cycle (8) (5) metabolism (4–7) demonstrating the wide range of VDR dependent genomic actions. Non-genomic actions include the activation of one or more intracellular signaling molecules, such as G protein-coupled receptors (G-protein), Protein kinase C (PKC), phosphatidylinositol-3′-kinase (PI3K), and Phospholipase C (PLC) resulting in opening of the voltage-gated channels, generation of the specific second messengers and cross talk with genomic pathways (8).

Dermal synthesis starts with the conversion of cutaneously derived cholesterol precursor 7-dehydroxycholestrol to previtamin D_3_ by the action of UV sunlight (44, 47). Previtamin D undergoes a temperature dependent isomerization to Vitamin D_3_. Vitamin D-binding protein (VDBP) carries the Vitamin D_3_ synthesized in the skin and Vitamin D_2_/Vitamin D_3_ absorbed via intestine from the dietary sources to the liver (45). In the liver, Vitamin D_2_/Vitamin D_3_ is hydrolyzed to 25-hydroxyvitamin D[25(OH)D] by the action of enzyme 25-hydroxylase (CYP2R1) (44). The final step of activation occurs in the kidney with the conversion of 25-hydroxyvitamin D[25(OH)D] to 1,25(OH)_2_D by the 1α-hydroxylase enzyme (CYP27B1) (48). Finally, 1,25(OH)_2_D binds to its principal receptor VDR through which it regulates the expression of a large number of genes across various tissues in the human body (46). VDR plays a central role in mediating the biological functions of Vitamin D, via both genomic and non-genomic pathways as elucidated in Figure 2.

## Vitamin D Status and Dosage

**Figure 3:**
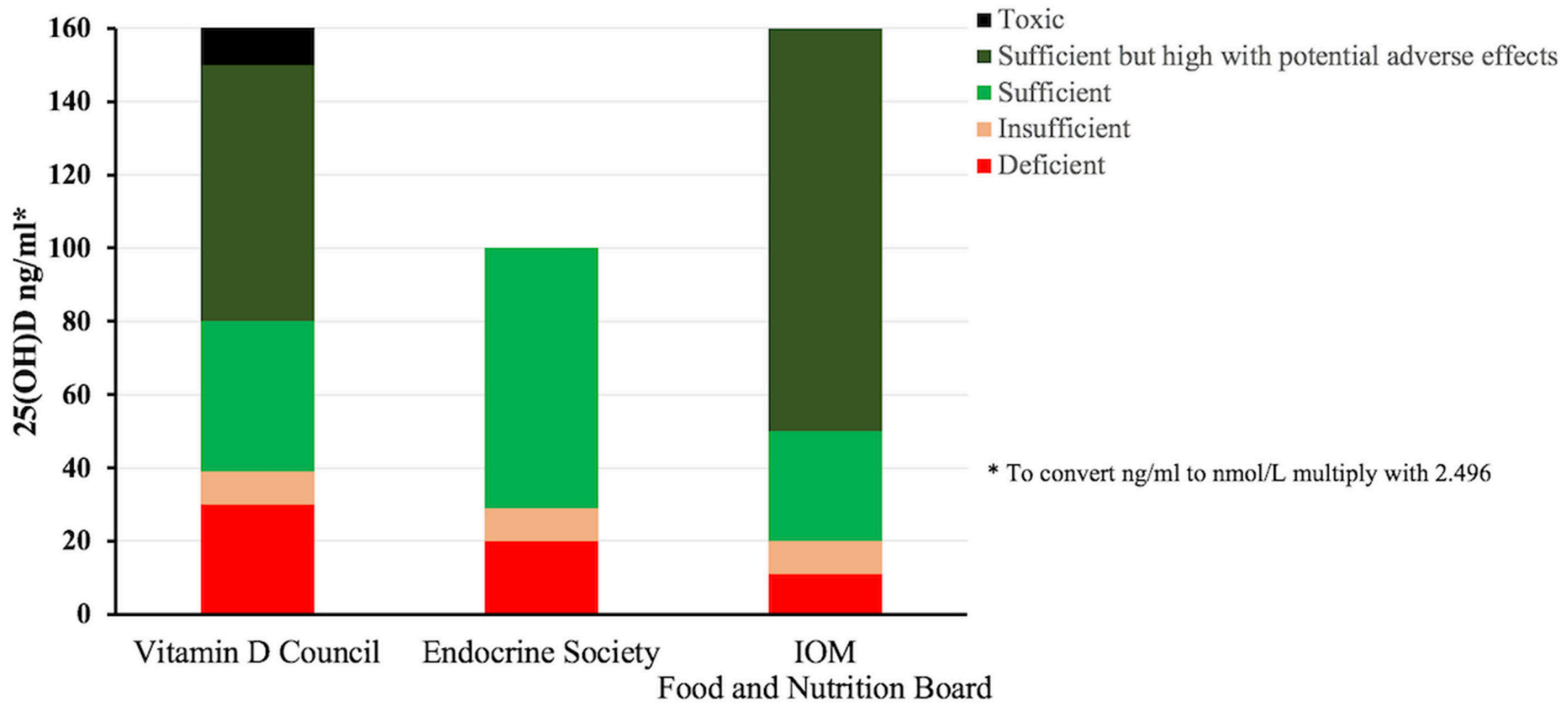
Vitamin D status classification according to different internationally recognized organizations. The lack of a consensus about the ranges for deficiency and sufficiency among various international groups is apparent, Vitamin D council (1) defines Vitamin D deficiency in the range of 0–30 ng/ml whereas Endocrine Society (2) and Institute of Medicine (3) defines it as 0–20 and 0–11 ng/ml, respectively. Similarly, sufficiency is defined as 40–80, 30–100, and >20 ng/ml by (1), (2), (3), respectively. Vitamin D serum levels measured above 150 ng/ml is considered toxic by (1) where as there is no such interpretation by (2) and (3) (50–52).

Levels of Vitamin D are measured in the serum as 25(OH)D, reflecting the Vitamin D status. The results of this blood test help with the clinical decision as to whether to take Vitamin D supplement or expose the skin to the sun. However, there is no international guidelines for reading the Vitamin D levels, as different organizations interpret the levels differently. Levels indicated as normal by one method may be interpreted differently by the other. The Vitamin D Council guidelines recommends 40–80 ng/ml as the ideal level, with levels of 0–30 ng/ml being considered deficient and 31–39 ng/ml considered insufficient (50). According to the Endocrine Society (51) the recommended preferred range falls between 40 and 60 ng/ml, levels below 20 ng/ml should indicate deficiency, 20–29 ng/ml defines insufficiency and ≥30 ng/ml is considered sufficient, all these guidelines along with the ones from the Food and Nutrition Board (52) are summarized in Figure 3.

Measurement of 25(OH)D can be performed with a number of different analytical techniques. Automated immunoassay's is used by most pathology lab to measure total serum 25(OH) D (25(OH) D_2_ + 25(OH) D_3_). Because of the clinical importance of Vitamin D testing it is important to note the existing discrepancies in Vitamin D total immunoassays as reported by several studies (53, 54). Various causes of the discrepancies have been noted (55) including the cross-reactivities of various Vitamin D metabolites (56). Therefore, the assays for Vitamin D testing need to be standardized so there is less variability in the results, the Vitamin D Standardization Program (VDSP), was launched in 2010 in collaboration with the National Institutes of Health, the Centers for Disease Control and Prevention (CDC), the National Institute for Standards and Technology (NIST), and Ghent University in Belgium to correct the disparity and ensure reliable Vitamin D measurement. However, we are still a long way to go to attain the goal of standardization of assay and results, till then we have to rely on the judgement of physicians and experts in the field. The researchers who publish in this area will likely continue to point toward such disparities.

To overcome the deficiency, Vitamin D supplementation is usually advised, however there is no consensus on the optimal dose to be prescribed, as many factors such as age and serum levels of 25(OH)D must be taken into account before recommending the best possible dose for instance for obese people (BMI > 30 kg/m^2^), a daily Vitamin D dose was set as “three times” greater than the recommended dose for subjects with normal body weight (51). The most common form of Vitamin D supplementation is Cholecalciferol and Ergocalciferol with both considered highly effective and safe (57). Vitamin D toxicity generally results from having serum levels of 25(OH)D > 150 ng/ml, which in most cases is attributed to prolonged and unintended daily intakes of >40,000 IU of supplementation (58). Table 1 details the group-wise recommendations for daily dose of Vitamin D supplementation from different organizations.

**Table 1:** Group-wise recommendations for daily dose of Vitamin D supplementation from different organizations.

	**Vitamin D council (IU/day)**	**Endocrine society (IU/day)**	**Food and nutrition board (IU/day)**
Adult	5,000	1,500–2,000	600
Infant	1,000	400–1,000	400
Children	1,000	600–1,000	600
Pregnant women	4,000–6,000	1,500–1,000	600

*Disparity in Vitamin D dosage recommendations by the Vitamin D council (1), Endocrine society (2), IOM food and nutrition board (3) are shown. Generally, however, pregnant or breastfeeding women and the elderly as well as children and adults lacking enough exposure to sunlight are in need of Vitamin D supplementation (50–52)*.

## Vitamin D Deficiency: a Health Problem Worldwide and in the GCC Countries

Vitamin D deficiency is a major public health problem worldwide, affecting more than a billion people across the globe from both the developing and industrialized countries (59, 60). Studies have suggested that more than 70% of USA and 50% of UK adults may have insufficient Vitamin D levels (61, 62). Among other European populations, 1 in 8 adults have low circulating levels of 25-hydroxyvitamin D (63) and a similar pattern has also been reported in India, Australia and New Zealand (64, 65).

**Figure 4:**
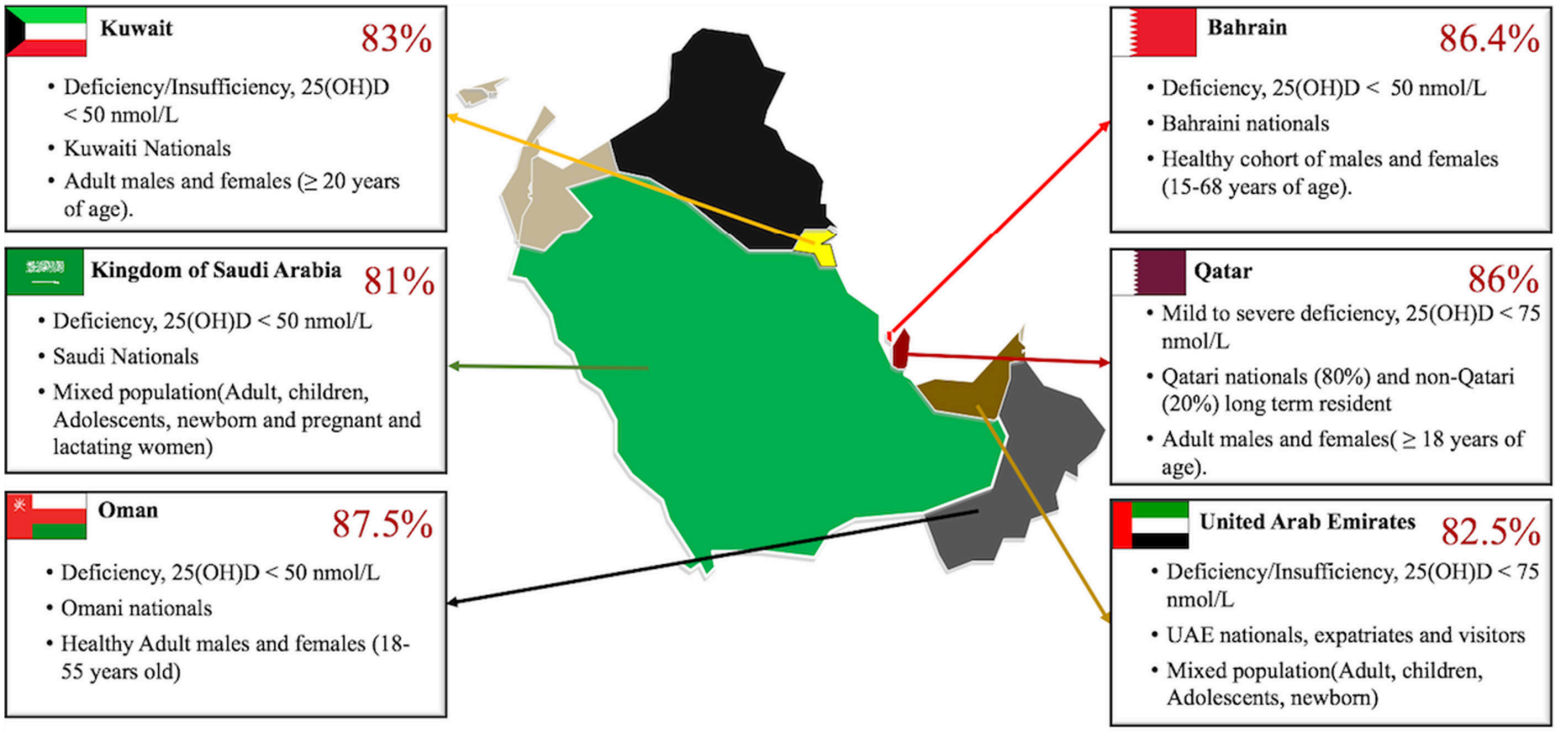
Vitamin D deficiency reported in GCC countries. Gulf Cooperation Council (GCC), alliance of six Middle Eastern countries—Saudi Arabia (KSA), Kuwait, the United Arab Emirates, Qatar, Bahrain, and Oman. The Vitamin D deficiency runs high in the multi-ethnic population of these countries with percentages as high as 86% in Qatar (66) other countries such as KSA (67), UAE (68), Oman (69), and Kuwait (70) follow closely similar alarmingly high rates of Vitamin D deficiency/Insufficiency. The GCC map used was obtained with copyright approval from: https://yourfreetemplates.com/free-middle-east-map/.

Despite the ample amount of year-round sunlight in the GCC region, pandemic levels of Vitamin D deficiency have been observed in recent years as represented in Figure 4. The 2016–2017 annual report from the Qatar Biobank highlights that close to 86% of the total Qatar biobank population (comprising of adults above 18–85 year-old, 80% of them Qatari national and the rest were long-term residents) suffers from Vitamin D inadequacy and more interestingly, 14% (more women 65% than men 35%) remained deficient despite taking supplementation, 70% of the participants were also obese (71). A review examining mixed population in Saudi Arabia (e.g., pregnant/ lactating women, children, adults) found that in 81% of all the groups the levels of 25(OH) D was <20 ng/mL (<50 nmol/L) (67). The numbers run high in UAE as well, a study conducted with a large cohort of patients (60,979) including UAE nationals and visitors of other nationalities, showed that up to 82.5% of the population suffers from deficiency or insufficiency of Vitamin D. Around 86.1% UAE nationals and 78.9% visitors had serum levels of 25(OH)D < 75 nmol/L. The study also showed that the extreme cases of deficiency were higher in females (26.4%) than males (18.4%) (68). Comparable percentages have also been reported in different study cohorts' in other countries in the gulf peninsula (69, 70).

Recently many studies have associated high levels of Vitamin D deficiency with immune-mediated and inflammatory diseases. IBD is the term used to describe disorders that involve chronic inflammation of the GI tract. The main types of IBD include: ulcerative colitis and Crohn's disease. Recently the Epi-IBD study reported high prevalence of low Vitamin D levels in treatment-naive European IBD populations, with 79% of the patients showing either insufficient or deficient levels of Vitamin D (72). Vitamin D supplementation was associated with reduced intestinal inflammation in patients with active UC (73) and also in controlling the relapse rate of IBD (74).

Systemic lupus erythematosus (SLE) is a chronic autoimmune inflammatory disease which involves the connective tissue effecting multiple organs such as the brain, lungs, kidneys, heart, blood vessels, muscles, skin etc., and is more common among women (75). Vitamin D deficiency was significantly higher in SLE patients when compared to healthy controls in a study conducted with a cohort of Bahraini patients (76). Similar results were observed in a Saudi cohort (71) with high prevalence of Vitamin D inadequacy was observed in Saudi patients with SLE.

T1DM prevalence/incidence is increasing worldwide (77) the rise seems much steeper in gulf countries. A study conducted to determine the association between Vitamin D status and T1DM along with other factors in the young population of the State of Qatar found out that the incidences of severe Vitamin D deficiency was considerably higher in T1DM (28.8%) compared with healthy children (17.1%) (78), similarly in the Saudi cohort 84% of the T1DM children, and 59% of the healthy children were Vitamin D deficient (79). Low serum Vitamin D status was found to be associated with high prevalence and early onset of type-1 diabetes mellitus in Kuwaiti children as well (80). In a case control study done with Multiple sclerosis patient in Kuwait, VDR variants *Taq*I and *Bsm*I were found to be associated with MS risk (81).The therapeutic effect of Vitamin D in immune diseases should be further assessed in interventional studies.

A comparative associative study between Vitamin D deficiency and IBS (Irritable bowel syndrome) was conducted with patients visiting the gastroenterology clinic in Saudi Arabia, Vitamin D deficiency was detected in 82% of the patients in the IBS group and 31% in the control group (82).

The potential limitation to the findings of this review should be considered, relatively small number of studies were available to be included in the review and also there is limited data to prove causal relationship between Vitamin D deficiency and risk of Immune system related illness. The studies included employ various definitions of hypovitaminosis D, and these variations may result in the over or underestimation of Vitamin D deficiency in GCC population. Thus, development of global or local standard and guidelines will help in better screening to define the candidates and treating those who are at most risk for Vitamin D deficiency.

As we previously discussed, synthesis of Vitamin D occurs in the skin and it depends on several factors such as time of sun exposure, season, latitude, altitude, clothing, veiling, use of sunscreen, old age, and skin color (83). It was previously shown that limited exposure to sunlight results in poor Vitamin D synthesis (84), thus even in sunny climates like the GCC countries, high rates of Vitamin D deficiency is found because of cultural and social habits that limit exposure to sunlight (85). The association between reduced 25D concentrations and obesity is also well-established with several large-scale studies that found obesity to be associated with lower 25D, 1,25D concentrations (86, 87). A bi-directional genetic study revealed that though the effect of lower 25D on BMI may not be significant but higher BMI leads to lower 25D (88) various explanations have been proposed to define this association, such as reduced cutaneous synthesis (89) and altered metabolism (90). Dietary sources make up for <10% of the total Vitamin D synthesized in the body (91), so even with a varied balanced diet, individuals will not be able to achieve the recommended Vitamin D levels.

However, the insufficiency of Vitamin D may not be solely due to diet or lack of sunlight. Several studies have suggested that various genes can define the Vitamin D status, thus large-scale genome-wide association studies have identified selected genes mainly those involved in the synthesis, metabolism or transport of Vitamin D to be associated with a variation in the Vitamin D status (92, 93). The SNPs associated with these genes were initially singled out in a European population (92) and were later replicated in the African American (94) and Asian populations (95). We utilized the GWAS catalog to generate the list of 49 gene variants or risk allele associated with Vitamin D Levels as shown in Table 2 below. After literature review we identified two gene-association studies conducted in the GCC region, one in the Saudi populations (104) and the other in the Kuwaiti population (105). The study performed in the Saudi population identified a significant risk of Vitamin D deficiency and insufficiency associated with the SNP rs2228570 [Chromosome: 12, Position: 47879112, Gene: Vitamin D Receptor (VDR)], rs4588 [Chromosome: 4, Position: 71752606, Gene: GC] and rs10741657[Chromosome: 11, Position: 14,893,332, Gene: Vitamin D 25-hydroxylase (CYP2R1)] (104), while the study conducted in Kuwait showed that the polymorphism in the GC gene coding for the Vitamin D Binding protein (VDBP) may play a major role in determining Vitamin D levels in this population (105).

**Table 2:** List of key variants or risk allele associated with Vitamin D level as reported by the GWAS catalog.

**Number**	**SNP**	**Reported gene**	**Region**	**Location**	**Functional class**	**Reported trait**	**References**
1	rs3755967-T	^*^GC (Group Specific Component)	4q13.3	4:71743681	Intron variant	Vitamin D levels	(96)
2	rs4588-T	GC (Group Specific Component)	4q13.3	4:71752606	Missense_variant	Vitamin D levels	(97)
3	rs2282679-T	GC (Group Specific Component)	4q13.3	4:71742666	Intron_variant	Vitamin D levels (dietary vitamin D intake interaction)	(96)
4	rs705117-G	GC (Group Specific Component)	4q13.3	4:71742398	Intron_variant	Serum vitamin D-binding protein levels	(98)
5	rs7041-T	GC (Group Specific Component)	4q13.3	4:71752617	Missense_variant	Serum vitamin D binding protein levels	(98)
6	rs2282679	GC (Group Specific Component)	4q13.3	4:71742666	Intron_variant	Vitamin D insufficiency	(92)
7	rs1607741-C	GC (Group Specific Component)	4q13.3	4:71853316	Intergenic_variant	Vitamin D levels	(97)
8	rs2282679-C	GC (Group Specific Component)	4q13.3	4:71742666	Intron_variant	Vitamin D levels	(99)
9	rs79761689-C	GC (Group Specific Component)	4q13.3	4:72005565	Intergenic_variant	Vitamin D levels	(97)
10	rs2282679-?	GC (Group Specific Component)	4q13.3	4:71742666	Intron_variant	Vitamin D levels	(100)
11	rs17467825-A	GC (Group Specific Component)	4q13.3	4:71739800	Downstream_gene_variant	Vitamin D levels	(101)
12	rs1155563-C	GC (Group Specific Component)	4q13.3	4:71777771	Intron_variant	Vitamin D levels	(101)
13	rs4588-A	GC (Group Specific Component)	4q13.3	4:71752606	Missense_variant	Serum 25-Hydroxyvitamin D levels	(102)
14	rs116970203-A	CYP2R1 (Vitamin D 25-hydroxylase)	11p15.2	11:14855172	Intron_variant	Vitamin D levels	(97)
15	rs10741657-A	CYP2R1 (Vitamin D 25-hydroxylase)	11p15.2	11:14893332	Upstream_gene_variant	Vitamin D levels	(96)
16	rs10741657-?	CYP2R1 (Vitamin D 25-hydroxylase)	11p15.2	11:14893332	Upstream_gene_variant	Vitamin D insufficiency	(92)
17	rs2060793-A	CYP2R1(Vitamin D 25-hydroxylase)	11p15.2	11:14893764	Upstream_gene_variant	Vitamin D levels	(99)
18	rs117913124-A	CYP2R1 (Vitamin D 25-hydroxylase)	11p15.2	11:14879385	Synonymous_variant	Serum 25-Hydroxyvitamin D levels	(102)
19	rs17216707-T	CYP24A1 (1,25-dihydroxyvitamin D(3) 24-hydroxylase)	20q13.2	20:54115823	Regulatory_region_variant	Vitamin D levels	(96)
20	rs6127099-T	CYP24A1 (1,25-dihydroxyvitamin D(3) 24-hydroxylase)	20q13.2	20:54114863	Intergenic_variant	Vitamin D levels	(97)
21	rs12785878-T	NADSYN1/DHCR7 (7-dehydrocholestrol reductase)	11q13.4	11:71456403	Intron_variant	Vitamin D levels	(96)
22	rs12785878-?	NADSYN1 (7-dehydrocholestrol reductase)	11q13.4	11:71456403	Intron_variant	Vitamin D insufficiency	(92)
23	rs4423214-T	NADSYN1(7-dehydrocholestrol reductase)	11q13.4	11:71462208	Intron_variant	Vitamin D levels	(97)
24	rs4944062-T	NADSYN1(7-dehydrocholestrol reductase)	11q13.4	11:71476248	3_prime_UTR_variant	Vitamin D levels (dietary vitamin D intake interaction)	(96)
25	rs3829251-A	NADSYN1 (7-dehydrocholestrol reductase)	11q13.4	11:71483513	Intron_variant	Vitamin D levels	(99)
26	rs182244780-A	RRAS2 (RAS related 2)	11p15.2	11:14363985	Intron_variant	Vitamin D levels	(97)
27	rs12287212-A	RRAS2 (RAS related 2)	11p15.2	11:14428315	Intergenic_variant	Vitamin D levels	(101)
28	rs11023332-C	PDE3B (phosphodiesterase 3B)	11p15.2	11:14762564	Intron_variant	Vitamin D levels	(101)
29	rs1007392-A	PDE3B (Phosphodiesterase 3B)	11p15.2	11:14753045	Intron_variant	Vitamin D levels	(101)
30	rs117300835-A	CALCB/INSC (Calcitonin Related Polypeptide Beta)	11p15.2	11:15097429	Intergenic_variant	Vitamin D levels	(97)
31	rs55665837-T	COPB1(Coatomer Protein Complex Subunit Beta 1)	11p15.2	11:14473503	Intron_variant	Vitamin D levels	(97)
32	rs148189294-A	SLC4A4(Sodium bicarbonate cotransporter 1)	4q13.3	4:71575200	Downstream_gene_variant	Vitamin D levels	(97)
33	rs117865811-G	SPON1(Spondin 1)	11p15.2	11:14180763	Intron_variant	Vitamin D levels	(97)
34	rs138485827-T	NPFFR2/ADAMTS3 (Neuropeptide FF Receptor2)/(A disintegrin and metalloproteinase with thrombospondin motifs 3)	4q13.3	4:72166226	Intergenic_variant	Vitamin D levels	(97)
35	rs78862524-A	ADAMTS3 (A disintegrin and metalloproteinase with thrombospondin motifs 3)	4q13.3	4:72305473	Intron_variant	Vitamin D levels	(97)
36	rs8018720-C	SEC23A	14q21.1	14:39086981	Missense_variant	Vitamin D levels	(96)
37	rs3819817-T	HAL (Histidine ammonia-lyase)	12q23.1	12:95984993	Intron_variant	Vitamin D levels	(97)
38	rs185378533-G	FLJ42102 (Uncharacterized LOC399923)	11q13.4	11:71422087	Intron_variant	Vitamin D levels	(97)
39	rs2207173-G	CYB5AP4(Cytochrome B5 Type A Pseudogene 4)	20p11.21	20:22824423	Intergenic_variant	Vitamin D levels	(103)
40	rs2277458-G	GEMIN2 (Gem Nuclear Organelle Associated Protein 2)	14q21.1	14:39114277	5_prime_UTR_variant	Vitamin D levels	(97)
41	rs10745742-T	AMDHD1(Amidohydrolase Domain Containing 1)	12q23.1	12:95964751	Intron_variant	Vitamin D levels (dietary vitamin D intake interaction)	(96)
42	rs12868495-A	VDAC1P12(Voltage dependent anion channel 1 pseudogene 12)	13q13.2	13:34067425	Intergenic_variant	Vitamin D levels	(101)
43	rs12144344-T	ST6GALNAC3(ST6 N-Acetylgalactosaminide Alpha-2,6-Sialyltransferase 3)	1p31.1	1:76373851	Intron_variant	Serum Vitamin D-binding protein levels	(98)
44	rs11586313-G	IVL (Involucrin)	1q21.3	1:152917994	TF_binding_site_variant	Vitamin D levels	(103)
45	rs6730714-A	PAX3 (Paired box gene 3)	2q36.1	2:222184302	Intergenic_variant	Vitamin D levels	(101)
46	rs156299-G	NPY (Neuropeptide Y)	7p15.3	7:24185113	Intergenic_variant	Vitamin D levels	(101)
47	rs2302190-C	MTMR4(Myotubularin Related Protein 4)	17q22	17:58507147	Missense_variant	Vitamin D levels	(101)
48	rs10508196-A	FAM155A (Family with sequence similarity 155 member A)	13q33.3	13:107827618	Intron_variant	Vitamin D levels	(101)
49	rs4751058-A	MKLN1 (Muskelin 1)	10q26.3	10:129075861	Intergenic_variant	Vitamin D levels	(101)

* *GC gene codes for the Vitamin D binding protein (VDBP)*.

## Vitamin D and the Gut Microbiome: is it a Bidirectional Relationship

The human gut microbiota (the microbial taxa associated with humans) is home to an estimated 10^14^ microorganisms, with around 500–1,000 species of bacteria (106). The vastness of the human microbiome (the catalog of human microbiota and their genes) can be imagined in terms that it contains 10 times more cells and 100 times more genes than the human (107). These microorganisms create a “mini-ecosystem” inside our bodies, and they work together as biochemical factories performing a wide array of activities such as acquisition of nutrients, Vitamins production, degradation of toxins and enhancing host immune responses by functioning as a barrier from pathogenic microorganisms (108).

Gut microbiota exists in a symbiotic relationship with the host. The composition of the gut microbiome plays a crucial role in maintaining host homeostasis, as the wrong combination of microbes contribute to an array of chronic digestive or immune disorders such as IBD, obesity, diabetes mellitus, metabolic syndrome, atherosclerosis, alcoholic liver disease, nonalcoholic fatty liver disease, cirrhosis, and hepatocellular carcinoma (109, 110).

**Figure 5:**
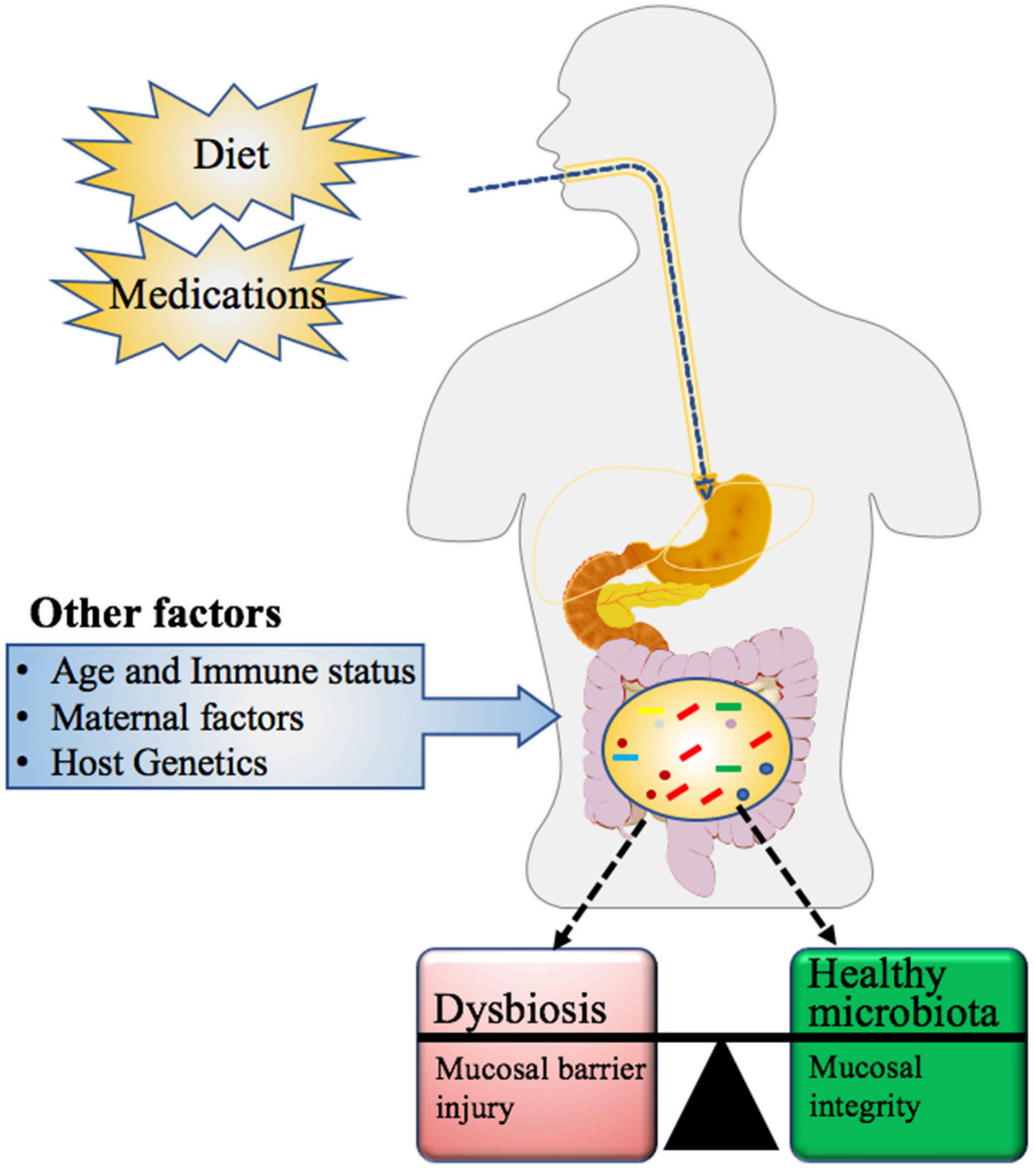
Factors influencing the microbial community composition in Humans. Maternal factors such as mother's nutritional status (use of pre or probiotics), mode of delivery (vaginal versus Cesarean section), gestation age at birth (full or preterm), feeding type (formula feeding or breastfeeding), and other exogenous factors such as diet and medication history, influence not only the gut microbial community but also its metabolic capacity. Other determinants of microbial composition include the age and immune health of the individual along with their genetic makeup (111).

The compositions of the gut microbiota is determined by several factors as summarized in Figure 5, which include age, host genomics, immune health, exercise, intake of medication specifically antibiotics, and dietary habits (111). This imbalance or maladaptation of microbial communities is often referred to as Dysbiosis (also called dysbacteriosis) which has been categorized into three different types: (1) loss of beneficial organisms, (2) excessive growth of potentially harmful organisms, and (3) loss of overall microbial diversity. These types can occur in combination and are not mutually exclusive (112, 113). In such cases, the normally dominant species become underrepresented and the resulting void is filled by increasing numbers of opportunistic or pathogenic species resulting in overall decrease in overall bacterial diversity (112). Dysbiosis is commonly reported in the gastrointestinal tract (112) and is associated with a loss of the integrity of the intestinal mucosa, which may result in gut impairment and inflammation (114–116).

**Figure 6:**
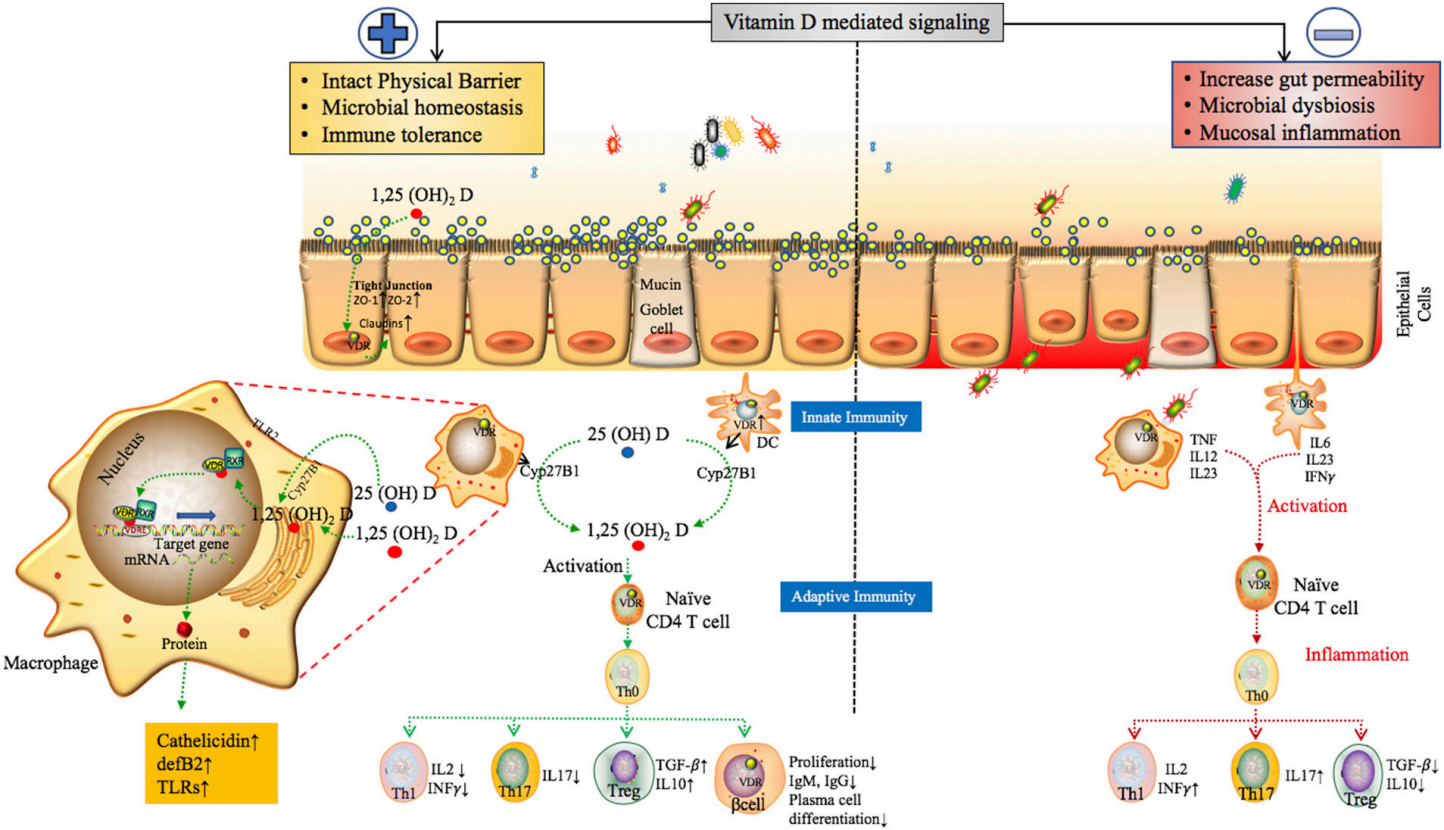
The proposed role of Vitamin D mediated signaling in epithelial cell barrier function, Gut microbial hemostasis and modulation of Innate and adaptive immune responses. VDR regulates the level of tight junction proteins ZO1, ZO2 via claudin2 and 12 (39, 118) and helps maintain the structural integrity of epithelial barrier. Vitamin D deficiency have also been shown to disrupt gut homeostasis leading to systemic inflammation (119). Effects of Vitamin D on different players of the innate and adaptive immune system. In response to an antigenic challenge, macrophages, dendritic cells, and lymphocytes express the Vitamin D receptor (VDR), thereby becoming targets for the active Vitamin D metabolite, 1,25(OH)_2_D (120). Macrophages and dendritic cells also express the CYP27B1 that synthesizes 1,25(OH)_2_D leading to intra and paracrine responses such as stimulates enhancing the phagocytotic responses of macrophages and production of antimicrobial proteins such as cathelicidin (49, 121, 122). Active Vitamin D also modulates adaptive immunity. At the level of the APCs (like DCs), 1,25(OH)_2_D inhibits the surface expression of MHC-II-complexed antigen. In addition, 1,25(OH)_2_D directly affects T cell responses, by inhibiting the production of Th1 cytokines (IL-2 and IFN-γ), Th17 cytokines (IL-17 and IL-21), and by stimulating Th2 cytokine production (IL-4) and Treg cell development (123–129). Finally, 1,25(OH)_2_D blocks plasma-cell differentiation, IgG and IgM production and B-cell proliferation (130). The above factors can trigger inflammatory immune responses such as TNF-α and IFN-γ leading to intestinal permeability and susceptibility to pathogenic infections, microbial dysbiosis and manifestation of immune related inflammatory diseases (19).

Several studies have demonstrated the role of Vitamin D and its receptor VDR in regulating host-microbial interactions. The active and inactive forms of Vitamin D circulate in the bloodstream bound to VDBP. The active form known as Calcitriol 1,25(OH)_2_D is known to bind to the calcitriol receptor, commonly known as the VDR (117). Calcitriol binds to the VDR, which then forms a heterodimer with the retinoid-X receptor and other co-activators. This transcriptional complex then binds to discrete sites on DNA known as Vitamin D responsive elements (VDREs) resulting in the expression or repression of specific gene products (summarized in Figure 6) (131). It is also known that Vitamin D plays a role in various microRNA-directed post-transcriptional mechanisms (132).

The Interplay between Vitamin D, VDBP, Cyp27B1, and VDR not only regulate the transcriptional and post-transcriptional responses, but a growing evidence supports the concept that Vitamin D metabolism impacts the intestinal microbial balance and gut homeostasis (132, 133). As previously mentioned, VDR is expressed in a variety of cell types such as kidney, muscles, prostate, immune cells; and high levels of expression in the cells of the gastrointestinal tract (GI) have been shown (134). Moreover, the Vitamin D activating enzyme Cyp27B1 is also expressed in various immune cells, as well as intestinal epithelial cells (135, 136). These two vital players of Vitamin D metabolism have also been colocalized in different cells of the GI tract suggesting the need for active Vitamin D in those cells (137).

In addition, the type of bacterial communities in the gut have been shown to regulate the expression of both VDR and Cyp27B1 (138, 139). VDR expression is inversely related to the presence of pathogenic bacteria, and correlate with probiotic bacteria with the former decreasing (138) and the latter increasing VDR expression (139). Animal studies done with germ-free and antibiotics-treated mice have reported reduced levels of Cyp27B1 expression in those animal models (140). This suggests that the intestinal bacteria regulate Vitamin D metabolism and, as a result, they regulate the innate immune response. The synthesis of active Vitamin D is modulated according to the local needs by Cyp27B1 and VDR enzymes whose expression is dependent on the “nature of local microbiota.”

The VDR, Cyp27B1, and VDBP genes have also been studied for their association with autoimmune diseases such as IBD and its forms Crohn's disease (CD) and Ulcerative colitis (UC) (72, 141, 142). Several VDR polymorphisms such as *ApaI, TaqI*, and *FokI* have been associated with the development of UC (143, 144). VDR and Cyp27B1 gene knockout mice have shown increased susceptibility to the development of intestinal colitis, elevated expression of proinflammatory cytokines, and microbial dysbiosis, with more *Proteobacteria* and less *firmicutes*, a condition commonly observed in patients with IBD (145). Vitamin D supplementation in human and 1,25D treatment of CYP27B1 knockout mice decreased *Proteobacteria* and increased beneficial organism including members of the *Firmicutes* phyla (132, 146).

Gut mucosal integrity is a crucial barrier, playing a vital role in protection against pathogenic microorganisms, as disruption in mucosal barrier function and hyperpermeability can predispose to various diseases such as asthma, tuberculosis, cystic fibrosis and intestinal lung diseases (147–149). Vitamin D and its associated molecules provide protection for the epithelial barriers in various tissues including the gut mucosa, by increasing the expression of several tight and adherent junction proteins (150–152). VDR signaling specifically has been known to regulate the gut mucosal cell inflammation by suppressing intestinal epithelial cell apoptosis (153). The increasing awareness of the role of Vitamin D as an immunomodulator was prompted by the discovery of VDR and production of active Vitamin D (1,25(OH)_2_ D) in almost all immune cells, including activated CD4+ and CD8+ T cells, B cells, neutrophils and antigen-presenting cells (APCs) such as macrophages and dendritic cells. The details of this regulation are described in the section below.

## Vitamin D in Innate and Adaptive Immune System

The innate immune system is the first and immediate line of the defense against invading pathogens and is an alliance of components both from the host and resident microbiota. The host defense comprises of diverse components such as physical defense (such as epithelial cells of the skin, mucous membrane, and microbiome), cellular defense (as mast cells, dendritic cells, macrophages, neutrophils, natural killer cells etc.), cell receptors that recognize pathogens (as Toll-like Receptors), antimicrobial peptides and proteins (as defensins, cathelicidins).

Vitamin D is able to modulate many of the above listed components of the innate immune system. It is known to reinforce the physical barrier function of epithelial cells. The active Vitamin D and VDR are important in regulating the genes of proteins required for the tight, adheres, and gap junctions such as zonulin occluden-1, zonulin occluden-2 through the up regulation of claudin 2 and 12 (154). Studies in transgenic mice demonstrated that over-expression of VDR in the intestinal epithelium decreases mucosal inflammation suppressing epithelial cell apoptosis and boosting the function of tight junction (119, 155). The importance of active Vitamin D in maintaining healthy gut microbiota has been discussed in the earlier section.

Monocytes and macrophages are crucial members of the innate immunity, Vitamin D stimulates the differentiation of precursor monocytes to mature phagocytic macrophage, the high expression of VDR by monocytes ensures sensitivity of these cells to the differentiating effects of active Vitamin D (120). Mature macrophages sense pathogen-associated molecular patterns (PAMPs) by means of pattern-recognition receptors, such as Toll-like receptors (TLRs). The presence of CYP27B1 (Vitamin D activating enzyme) in macrophages is important for the physiological action of host defense against infection, activation of 25(OH) D to 1, 25(OH)_2_ D via CYP27B1 in macrophages leads to regulation of TLR2 (121), enhanced production of defensin β2, cathelicidin antimicrobial peptide (CAMP) (49) leading to induction of autophagy (122). The ability of 1,25(OH)_2_D to increase the production of other antimicrobial peptides, has been demonstrated both *in vitro* by monocytes stimulation (156) and *in vivo* in pediatric patients' blood (157).

At the same time, the active Vitamin D can inhibit the dendritic cell (DCs) differentiation and maturation. In human and murine DC cultures, the activation of VDR signaling pathways inhibited DC-maturation as shown by downregulation of DC markers, MHC-class II, co-stimulatory molecules (CD40, CD80, and CD86), and other maturation molecules (e.g., CD1a, CD83), chemokine (CXCL10) which is involved in the recruitment of T helper 1 (Th1) cells. Furthermore, active Vitamin D also modulates DC-derived cytokine and chemokine expression, by inhibiting the production of pro-inflammatory cytokine (IL-12 and IL-23) and enhancing the release of anti-inflammatory cytokine (IL-10) and the chemokine [CCL22, involved in the recruitment regulatory T cells (Tregs)] (123–125).

However, this interaction between active Vitamin D and cells from innate immunity also have downstream effects on cells from the adaptive immune system. The adaptive immune system or acquired immune system is the second line of defense against infection. It comes into action upon exposure to pathogens and uses immunological memory to learn about the pathogens and enhance the immune response accordingly. The adaptive immune system is composed of T and B cells and is also responsible for autoimmune reaction. 1,25(OH)_2_ D suppresses the immune responses mediated by Type 1 T helper (Th1) cells, by inhibiting the inflammatory cytokines IL-2 and interferon gamma (IFNγ) (125, 126) On the other hand, it has been reported to enhance cytokines associated with Th2 cells (127). The 1,25(OH)_2_ D also regulate Treg cells (known for their role in the inhibition of inflammation) by induction of Foxp3 (the transcription factor involved in the development and function of Treg cells) (128). In addition to Th1, Th2, and Treg cells (subsets from the CD4 + T cell lineage) there are distinct subset termed Th17 cells that produce IL-17, which has been implicated in the pathogenesis of a number of autoimmune diseases. Th17 is inhibited by 1,25(OH)_2_ D (129). 1,25(OH)_2_ D prevents the activation of nuclear factor kappa-B (NF-κB), a component of proinflammatory signaling pathway at the level of transcription [VDR/RXR has been reported to bind to NF-κB promotor (IL-12p20) thus preventing its activation] (158).

The proliferation of VDR-expressing B-cells is also suppressed by active Vitamin D negatively impacting Ig production and also inhibiting the differentiation of plasma cells and class-switched memory cells highlighting a potential role for Vitamin D in B-cell-related disorders such as SLE (130). Figure 6 details the proposed model of the overall impact of Vitamin D in the regulation of the epithelial barrier function, gut microbiota, and the immune system.

The above data elucidate the role of Vitamin D/VDR signaling in not only maintaining the microbial landscape in the gut in check and keeping the gut mucosal integrity intact but also as a potent immunomodulator. However, to fully understand the extent to which Vitamin D impact these complex systems, the multidirectional interaction between them needs to be considered. The importance of the interplay presents an encouraging field for further research with potential clinical implications.

## Discussion

Vitamin D deficiency is a major health problem in GCC countries including Qatar, indicating its importance as a regional and national health problem (67–71). Vitamin D deficiency affects various age groups and threatens the GCC populations with increased prevalence for chronic diseases such as IBD, cancer, diabetes among others. The reasons for the deficiency include limited exposure to sunlight, full body covering and low consumption of food rich in Vitamin D (83–85). The deficiency is treated with Vitamin D supplementation or intramuscular injections, however, despite being treated in a similar manner; there are interracial and interindividual variations in response to Vitamin D intake (159). The differences in serum Vitamin D levels among diverse ethnic groups have been previously reported in GWAS studies that have identified SNPs associated with genes from the Vitamin D pathway (92–95). These studies have indicated that certain ethnic groups may be predisposed to lower serum Vitamin D levels. We believe the same may be true for the population residing in GCC countries. These genetic variations may also impact the response to Vitamin D supplementation in these populations.

Originally known to cure rickets, our understanding on how Vitamin D impacts human health has broadened. The discovery of VDRs and Vitamin D associated enzymes in a variety of cells including cells of the innate and adaptive immune system was crucial in appreciating its role as a potent immunomodulator. Immune cells are not only a target for Vitamin D, but are also able to activate the hormone locally, thus postulating for an autocrine and paracrine role for the active Vitamin D (160). Inadequate amounts of Vitamin D could be linked to defective functioning of the associated autocrine and paracrine circuits eventually leading to various immune abnormalities.

Among other extra-skeletal effects, we have also come to realize its role in maintaining a healthy gut microbiota. The GI tract is home to a plethora of microorganisms that program the immune system preventing colonization by harmful bacteria and viruses. Several studies referenced in this review have suggested that Vitamin D signaling influences the microbial load and composition and ensures adequate innate and adaptive immune responses to pathogenic threats; though the questions still linger about the bidirectional and multidirectional interaction between these intricate systems. Vitamin D deficiency may result in abnormal immune functions causing inflammation, which, when unresolved, may lead to chronic conditions.

In conclusion, Vitamin D signaling mediated by key players, such as VDR, VDBP, and Cyp27B1, has a diverse physiological impact, enhancing the intestinal barrier function and contributing to enteric homeostasis. Studies have shown that low serum Vitamin D levels might be linked to several diseases and given the fact that the Vitamin D status is a modifiable factor, the potential therapeutic benefits from Vitamin D supplementation in the prevention of various diseases and maintenance of a healthy microbiota are undeniable. Therefore, the widespread Vitamin D deficiency in the GCC countries demands discussion about including Vitamin D testing as part of routine clinical care practices.

## Author Contributions

All authors listed have made a substantial, direct and intellectual contribution to the work, and approved it for publication.

### Conflict of Interest Statement

The authors declare that the research was conducted in the absence of any commercial or financial relationships that could be construed as a potential conflict of interest.
